# China’s public health initiatives for climate change adaptation

**DOI:** 10.1016/j.lanwpc.2023.100965

**Published:** 2023-11-15

**Authors:** John S. Ji, Yanjie Xia, Linxin Liu, Weiju Zhou, Renjie Chen, Guanghui Dong, Qinghua Hu, Jingkun Jiang, Haidong Kan, Tiantian Li, Yi Li, Qiyong Liu, Yanxiang Liu, Ying Long, Yuebin Lv, Jian Ma, Yue Ma, Kinay Pelin, Xiaoming Shi, Shilu Tong, Yang Xie, Lei Xu, Changzheng Yuan, Huatang Zeng, Bin Zhao, Guangjie Zheng, Wannian Liang, Margaret Chan, Cunrui Huang

**Affiliations:** a Vanke School of Public Health, Tsinghua University, Beijing, China; b School of Public Health, Key Lab of Public Health Safety of the Ministry of Education and National School of Public Health, Health Commission Key Lab of Health Technology Assessment, Fudan University, Shanghai, China; c Department of Occupational and Environmental Health, School of Public Health, Sun Yat-Sen University, Guangzhou, China; d Shenzhen Center for Disease Control and Prevention, Shenzhen, China; e State Key Joint Laboratory of Environment Simulation and Pollution Control, School of Environment, Tsinghua University, Beijing, China; f National Institute of Environmental Health, Chinese Center for Disease Control and Prevention, Beijing, China; g Public Meteorological Service Centre, China Meteorological Administration, Beijing, China; h National Institute of Infectious Diseases at China, Chinese Center for Disease Control and Prevention, Beijing, China; i School of Architecture, Tsinghua University, Beijing, China; j School of Climate Change and Adaptation, University of Prince Edward Island, Prince Edward Island, Canada; k Center for Global Health, School of Public Health, Nanjing Medical University, Nanjing, China; l School of Public Health, Queensland University of Technology, Brisbane, Australia; m School of Economics and Management, Beihang University, Beijing, China; n School of Public Health, Zhejiang University, Hangzhou, China; o Shenzhen Health Development Research and Data Management Center, Shenzhen, China; p Department of Building Science, School of Architecture, Tsinghua University, Beijing, China

**Keywords:** Climate change, Adaptation, Temperature, China, Sustainable development, Public health, Early warning system, Green space, One health, Environmental engineering, Vulnerability analysis, Indoor, Healthy city, Carbon neutrality, Health co-benefits, Health policy

## Abstract

China’s health gains over the past decades face potential reversals if climate change adaptation is not prioritized. China’s temperature rise surpasses the global average due to urban heat islands and ecological changes, and demands urgent actions to safeguard public health. Effective adaptation need to consider China’s urbanization trends, underlying non-communicable diseases, an aging population, and future pandemic threats. Climate change adaptation initiatives and strategies include urban green space, healthy indoor environments, spatial planning for cities, advance location-specific early warning systems for extreme weather events, and a holistic approach for linking carbon neutrality to health co-benefits. Innovation and technology uptake is a crucial opportunity. China’s successful climate adaptation can foster international collaboration regionally and beyond.

**Key message**

China risks losing health gains within a single generation if society fails to adapt to climate change. Over the past five decades, China has made remarkable progress in improving population health through poverty eradication, better nutrition, disease prevention, improved healthcare access, and health education. Ensuring successful adaptation to climate change is the next crucial challenge to preserve these hard-won health gains. Alarmingly, China’s temperature increase trajectory outpaces the global average, largely attributed to ecological shifts and the amplification by urban heat islands.^1^ This underscores the heightened urgency to prioritize public health protection for climate change adaptation, all while championing sustainable development. The adverse health implications of escalating temperatures, shifting rain patterns, and intensifying extreme weather episodes necessitate immediate and concerted actions. Effective adaptation requires recognizing China’s unique challenges and opportunities in urbanization, a growing burden of non-communicable diseases, an aging population, and risks of future pandemics. To mitigate these risks, successful adaptation must include green cities, better indoor environments, enhanced systems for alerting the public about dangerous weather phenomena and adopting a co-benefits approach that considers human health for carbon neutrality, green city designs, and lower barriers for new technology adoption. Innovative technology, like cooling towers and district distribution systems, offers energy-efficient solutions, while strategies such as increasing surface reflectivity play a role in curbing the urban heat island effect. Embracing big data, sensors, and the internet-of-things will be instrumental for the management of climate change adaptation. Yet, it is also essential to comprehend obstacles that hinder technology uptake, especially in places with limited resources. The achievement of successful climate adaptation is a shared global asset that can promote new international cooperation between China and the world.

## Introduction of climate change and health in China

### China’s geographical and population diversity in the context of adaptation

**Fig. 1:**
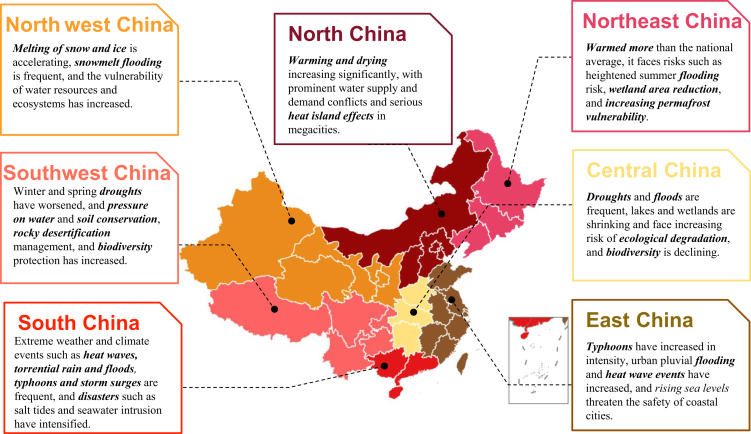
Map of regional extreme weather risks in China.

China‘s vastness and complexity defy a singular approach to climate adaptation. The nation’s rich tapestry of geographical features, from the icy reaches of the Tibetan Plateau and the towering Himalayas to the arid expanses of the Tarim Basin and the fertile North China Plain, introduces a set of varied challenges. A noticeable manifestation of the warming climate is the retreating glaciers on the Tibetan Plateau, a critical freshwater source for pivotal rivers like the Yangtze, Yellow, and Mekong.^2^ Further complicating matters, climate-induced erratic rainfall patterns have intensified both floods and droughts. While this geographical diversity presents its set of challenges, China’s demographic distribution adds another layer of complexity. The majority of the Chinese population is clustered in the eastern coastal regions, delineated by the *Hu Line* or *Qinling-Huaihe* Line (Box 1).^3^ East of this demarcation, major urban hubs such as Beijing, Shanghai, Guangzhou, and Shenzhen accommodate nearly two-thirds of the country’s population and are economic powerhouses. Fig. 1 provides a detailed depiction of risks across this vast landscape. Such intricate interplay between geography and demographics amplifies climate-induced health risks.^4^ Thus, it’s imperative to craft adaptation strategies that are finely tuned to the unique demands of each region and its inhabitants.

Box 1 **Population and temperate zones of China.**

Contrast in population density is represented by the ‘Hu Line’, a demographic boundary dividing the sprawling but underpopulated western territories from the densely inhabited eastern regions.

Temperate zones of China.

Adapted from Pulsipher, L.M. & Pulsipher, A. A. (2014, Jan 6). World Regional Geography without subregions: Global Patterns, Local Lives. New York: W. H. Freeman and Company.

### Mitigation and adaptation commitments under the UNFCCC framework

**Fig. 2:**
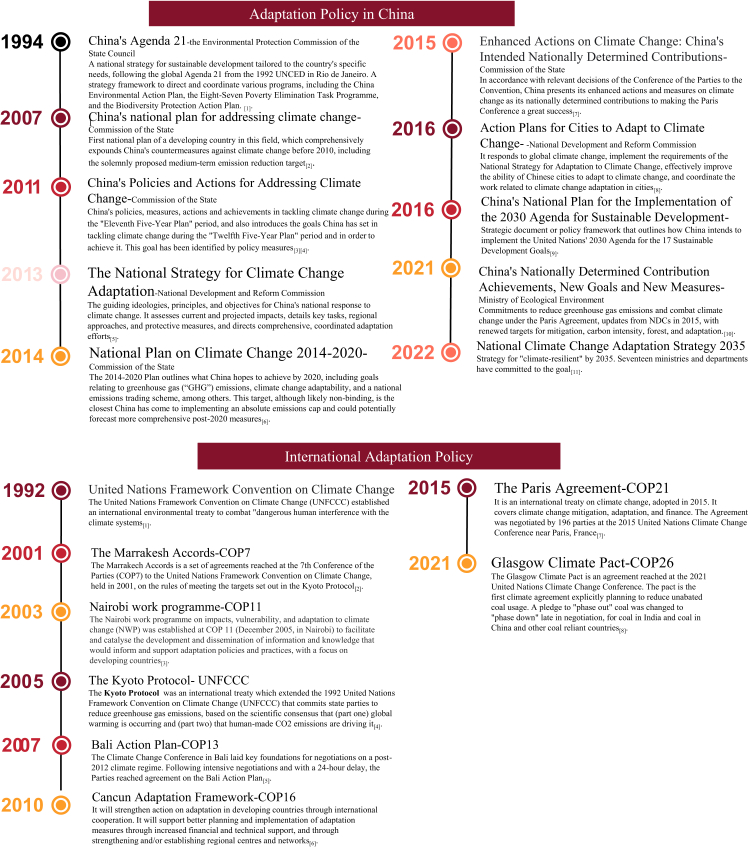
National and international adaptation policy timeline.

Mitigation takes precedence in China’s climate change policies, aiming to reduce reliance on fossil fuels by promoting renewable technologies and overcoming technological barriers. As an active participant in the United Nations Framework Convention on Climate Change (UNFCCC), China has submitted two rounds of Nationally Determined Contributions (NDCs).^5^ These commitments outline the country’s efforts to lower greenhouse gas emissions based on its national circumstances, capabilities, and priorities. China’s NDC includes targets to peak carbon dioxide emissions before 2030 and achieve carbon neutrality by 2060.^6^ Additionally, China acknowledges the importance of adaptation measures to address climate change impacts. Box 2 presents China’s Nationally Determined Contribution Targets and Potential Adaptation Co-benefits, recognizing the vulnerability of its ecological system, economy, and society. While mitigation remains a primary objective, policymakers also prioritize climate change adaptation, which involves adjusting to the impacts of climate change, reducing vulnerability, and building resilience. Fig. 2 illustrates the national and international timeline of adaptation policies. China’s NDC outlines specific measures to strengthen adaptation in sectors such as agriculture, water resources, and infrastructure, as well as enhance early warning systems and disaster risk reduction.

Box 2 **China’s nationally determined contribution targets and their associated mitigation co-benefits.**

**Table:** 

Increase the share of non-fossil fuels in primary energy consumption to around 25% by 2030	Non-fossil fuel sources have lower emissions of air pollutants compared to fossil fuels, leading to improved air quality and associated health benefits. Air pollution is currently a top risk for disease burden in China.
Increase forest coverage by 6 billion cubic meters from 2005 levels by 2030	Forests provide essential ecosystem services, including carbon sequestration, water regulation, and biodiversity conservation. Increasing forest coverage enhances desert regions to stabilize sand dunes and reduce the likelihood of dust storms and climate risks. Additionally, regional cooperation initiatives have been established to combat desertification and promote sustainable land management practices.
Increase the capacity of carbon sinks by 4.5 billion tons from 2005 levels by 2030	Carbon sink expansion often involves protecting or restoring ecosystems, which supports biodiversity conservation efforts, which are essential for maintaining ecosystem resilience and adaptive capacity. Trees, for example, provide shade and evapotranspiration, which can help cool the surrounding environment and reduce the urban heat island effect.

### National Strategy for Climate Change Adaptation 2035

Adaptation, in the context of China’s urban policies, should be defined as strategic measures specifically designed to address health risks posed by environmental changes. It requires a synergistic approach with mitigation to prevent exacerbating negative impacts. National Strategy for Climate Change Adaptation 2035 includes more concrete and comprehensive provisions related to the health risks of climate change. In the academic and policy communities, this strategic document represents a major step forward compared to the previous strategy issued in 2013 and replaces the earlier strategy that covered the period 2013–2020 and aims to make China’s society and economy more resilient to climate change. The new strategy recognizes that the global average temperature has increased and that temperature in China has increased at a faster rate of 0.26 °C per decade from 1951 to 2020 compared to the global rate of 0.15 °C per decade since the middle of the 20th century.^7^

### Climate adaptation in public health strategies

The Chinese government has seamlessly incorporated climate adaptation and public health considerations into numerous strategic plans. While there can be challenges in inter-governmental and inter-agency collaborations, stemming from resource constraints, diverse objectives, and organizational frameworks, China’s commitment remains evident. Prime examples are the Healthy China 2030 Plan, which prioritizes reduced air pollution and enhanced access to clean drinking water. Furthermore, the 14th Five-Year Plan (2021–2025) underscores the importance of health in policymaking, disease prevention, and the promotion of healthy lifestyles. This holistic approach extends to plans to emphasize ecological monitoring, pollution management, waste minimization, and alignment with global standards. Endeavors such as the Air Pollution Prevention and Control Action Plan and the Blue-Sky Defense Battle underscore China’s unwavering resolve to enhance air quality and environmental health. Collectively, these initiatives showcase China’s proactive stance in tackling climate change while advancing public health through well-defined policies and actions.^8^

## Climate risks and health impacts in China

### Compound events

In China, compound events are the occurrence of multiple extreme climate phenomena either simultaneously or in close time sequence. Unlike typical climate events, these have heightened repercussions for public health responses. The summer of 2023 in Beijing and neighboring Hebei and Tianjin areas have been battered by severe climate events, with the city grappling with a historically severe heatwave followed by a lethal flash flood due to Typhoon Doksuri (Supplementary Box S1 shows the Case Study 2023 Typhoon Doksuri). With climate change, such events are witnessing a rise in both frequency and intensity across diverse Chinese regions. The combined effects of these events magnify threats to human health and the nation’s infrastructure, making disaster mitigation, response, and rehabilitation more intricate.^9^

### Deforestation and sandstorms

Severe sandstorms are worsened by rising industrial activities and rapid deforestation throughout northern China. In recent years, severe sandstorms swept through Beijing and several northern provinces in China, sending air pollution soaring to hazardous levels. The air quality index exceeded monitoring upper limits, reflecting many orders of magnitude higher than the daily average guideline set by the World Health Organization.^10^ In response to these seasonal severe sandstorms, nearly a dozen provinces have issued ‘yellow’ warning (see below on early warning system). These sandstorms, originating from Mongolia, have gradually shifted towards central and eastern China, driven by a combination of low rainfall and low-pressure winds.

### Particulate matter, nitrogen oxides, ozone, and heat waves

Combined environmental exposures, encompassing particulate matter air pollution, ozone, NO_2_, and heat waves, can have synergistic impacts on public health. The health implications of these exposures, however, vary based on the specific components and their concentrations. While there is consensus about the detrimental effects of PM_2.5_ exposure, the causal relationship between certain air pollutant components and mortality remains debated. For instance, research in China has established a strong link between PM_2.5_ exposure and premature mortality, but the adverse impacts of NO_2_, an indicator of traffic-related pollution, become pronounced primarily in the colder regions of northeast China. This suggests that the health effects of pollutants can be modulated by regional climatic conditions. Differing regional characteristics further amplify variations. Northern China, with its heavy industries, tends to have higher concentrations of particulate matter, while southern China sees heightened ozone exposure due to its geographical and climatic conditions. The densely populated eastern region, particularly during specific events like the 2013 Shanghai heatwave, faces combined environmental challenges. In this event, temperatures soared above 40 °C (104 °F), exacerbating the photochemical reactions that produce smog.^11^ This led to a surge in tropospheric ozone levels. The confluence of such extreme heat and high ozone concentration not only posed immediate health threats but also heightened the risk of respiratory and cardiovascular diseases.^12^

### Cyclones, flood and disaster management

A study conducted in 153 counties in China found that exposure to tropical cyclones increased the risk of both accidental and non-accidental mortality, with circulatory and respiratory disease showing an 8% increase in non-accidental mortality on tropical cyclone-exposed days.^13^ Since 1990, along the Chinese coast, tropical cyclones with lower translation speed and higher rainfall totals have become more frequent,^14^ leading to over 60% of the largest hazardous floods in the northern and eastern parts of China.^15^ These findings highlight the need for long-term planning and preventive control of extreme weather events, as well as the development of health protection advice and early warning mechanisms for compound climate events.

### Climate-sensitive chronic diseases

Climate change acts as an amplifier of the health challenges already faced by China. Direct climatic factors, like extreme temperatures, play a pivotal role in exacerbating existing health conditions rather than initiating new cases of chronic diseases. Early phases of a heatwave, particularly the first two days, show more pronounced health impacts, emphasizing the importance of swift interventions.^16^ Notably, a study in 2017 highlighted that heatwaves significantly spiked mortality rates from cardiovascular diseases in China, with an increase of 27.8% on heatwave days compared to non-heatwave days.^17^^,^^18^

Dealing with climate change adaptation extends beyond just heat waves but also includes cold. Cold mortalities dominate the total non-optimal temperature mortalities, with the estimation that 593,900 thousand deaths were attributable to non-optimal temperatures in China in 2019 (PAF = 5·58%), with 580,800 cold-related deaths and 13,900 heat-related deaths. Cardiovascular diseases and chronic respiratory diseases were the leading causes of temperature-related deaths. The death rates for both high and low temperatures varied significantly across regions, with Western China having higher age-standardized death rates for low temperatures and Xinjiang and Central-Southern China having greater death rates for high temperatures.^19^ It is poorly understood whether climate change lowers cold-related mortality, or causes more cold-related mortalities with higher temperature fluctuations.

### One Health

**Fig. 3:**
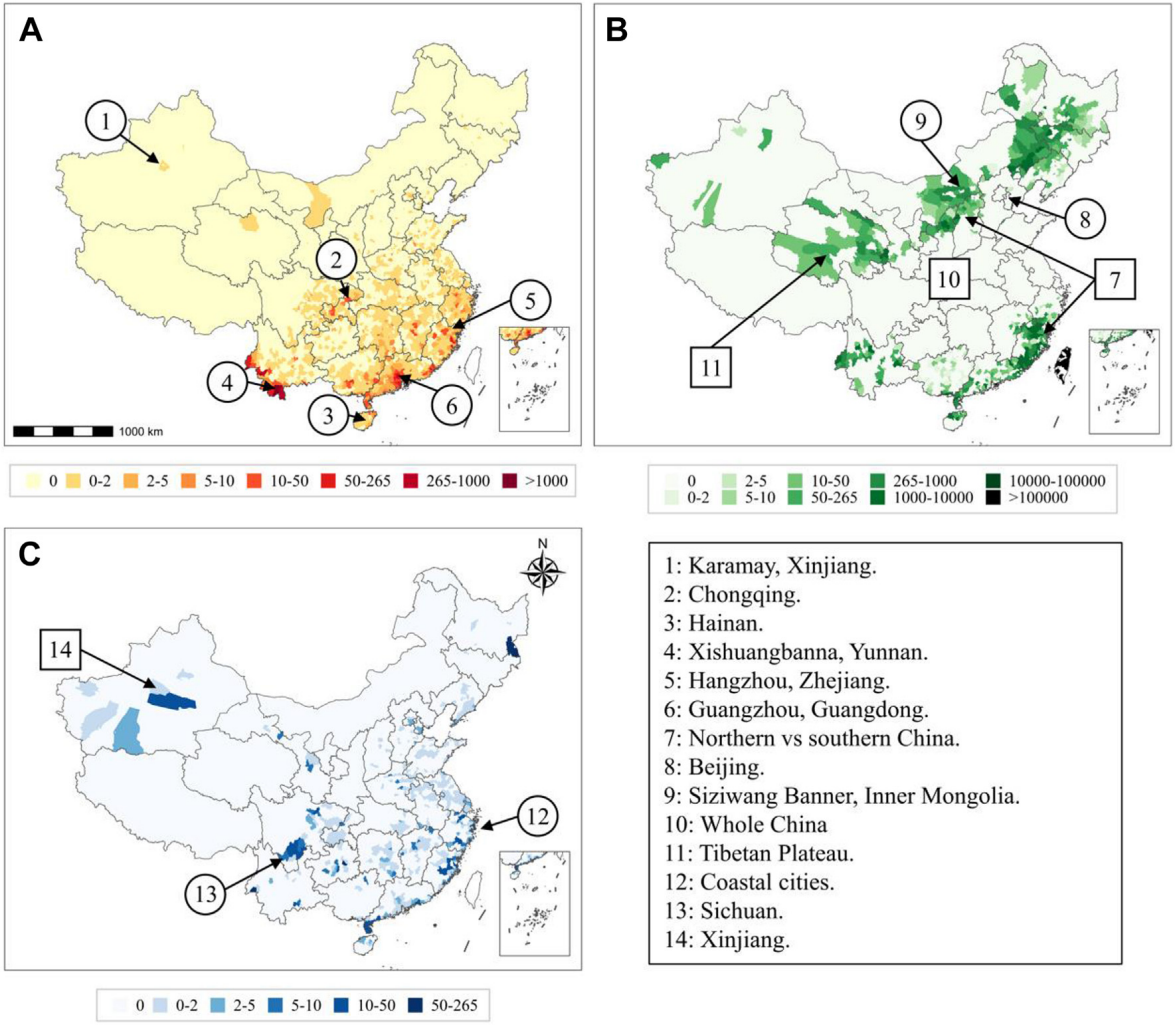
The cumulative incidence of 3 infectious diseases in China at county scale (100,000 persons). The map indicates the areas where infectious diseases have had severe outbreaks previously or may potentially occur in the future. Circles represent cities/provinces, boxes represent broader regions. (A) The vector-borne disease: dengue (2005–2020). (B) The rodent-borne disease: plague (1772–2019). (C) The water-borne disease: cholera (1999–2008). Evidence shows that infectious diseases are prone to be impacted by climate change, further explanation can be found in Supplemental file (Supplementary Web text S1).

One Health, defined by the World Health Organization (WHO), emphasizes the interconnected health of people, animals, and the environment. This approach is essential for managing zoonoses like dengue, plague, Hemorrhagic Fever with Renal Syndrome (HFRS), and cholera, as well as issues like food safety and antimicrobial resistance. Climate change, particularly in China, affects the transmission of these diseases, with changing weather patterns facilitating disease vectors such as mosquitoes and ticks.^20^ Fig. 3 presents the cumulative incidence of these three diseases in China per 100,000 individuals. Research has identified a strong connection between soil attributes and the emergence of plague reservoirs. Areas with high concentrations of metals such as cadmium, copper, iron, magnesium, sodium, antimony, and uranium, as well as elevated soil pH, are more susceptible to plague reservoir formation. In contrast, increased levels of calcium, cerium, molybdenum, and yttrium decrease the likelihood. The pH of the soil plays a pivotal role, in influencing the development of *Yersinia pestis* and flea larvae and affecting metal availability. Furthermore, high elevations, like those found in the Tian-Shan, Pamir, and Altai ranges and the Qinghai–Tibet plateau, are notable hotspots for plague reservoirs.^21^ Climate variables, particularly temperature and precipitation, indirectly modulate rodent and flea population behaviors, which can influence the spread and persistence of the plague. For effective climate change adaptation, understanding these relationships can be used to anticipate and manage potential plague outbreaks.

### Mosquito-borne infectious diseases

Mosquito-borne diseases are one of the climate-sensitive infectious diseases dramatically affected by climate change, the most prevalent of which is dengue fever.^22^ With 100–400 million new infections each year, it is one of the fastest-growing infectious diseases globally and is taking hold in many large tropical cities.^23^ Dengue outbreaks in China have spread from the southern coastal regions, including Hainan and Guangdong, to the northern and western regions of Zhejiang, Fujian, and Yunnan, with short outbreak intervals since 1990.^24^ A number of traditional preventive actions have been undertaken, including dengue vaccines, all of which are primarily live. Although there is an urgent need for a vaccine to prevent dengue, it is controversial that until now, no dengue vaccine has been widely used, and the ideal vaccine that can serve as effective prevention in large populations is under investigation.^25^ In addition, based on the limited effectiveness of traditional insecticide-based control behaviors, biological control measures such as Wolbachia are positioned as promising potential for dengue control intervention.^26^ In the near future, numerous studies predict an expansion of infected populations, driven by growing populations in endemic areas and changes in climate.^27^

### Rodent-borne diseases

Rodent-borne diseases in China, such as plague and Hantavirus hemorrhagic fever with HFRS, are notably influenced by climate change. Plague, caused by *Y. pestis*, is a zoonotic disease, while Hantavirus hemorrhagic fever with HFRS results from hantaviruses. Humans contract plague through contact with infected animals or their parasites, while HFRS transmission occurs via inhalation of contaminated aerosols from vectors like feces or saliva of infected animals.^28^ Climate alterations modify species ranges, affecting disease spread and human interaction. Mitigating these diseases focuses on managing natural habitats and limiting exposure to infected animals, like controlling rodent populations near human areas.^29^ Effective control mandates updated early warning systems and a comprehensive surveillance network combining case reports and laboratory findings.^30^

### Water-borne diseases

Climate change impacts water-borne diseases in China, with cholera, caused by *Vibrio cholerae*, being a prime example. The changing climate can amplify the spread of such pathogens through contaminated food and water sources. Since the 1990s, due to enhanced environmental management, cholera, while monitored, is no longer a predominant concern. Climate change adaptation strategies, including upgrading infrastructure, promoting better sanitation, and educating the public, are crucial in managing these waterborne diseases. Furthermore, we must be attentive to other agents causing gastroenteritis, which can also be swayed by the evolving climate. Monitoring at urban water purification plants now includes a range of pathogens, such as the novel coronavirus, Type A influenza virus, norovirus, rotavirus, and Salmonella, that are known to exhibit gastrointestinal excretion patterns.^31^^,^^32^

### Climate change impacts on health via labor productivity impacts

Climate change has a significant impact on labor productivity in China, in addition to its negative effects on health. Extreme temperatures not only result in medical expenses^33^^,^^34^ and mortality losses^35^ but also cause productivity losses,^36^^,^^37^ all of which contribute to a decline in gross domestic product (GDP). For instance, economists estimate that the 2017 exceptional heatwave in China led to an estimated economic loss of 61,304 million RMB due to premature deaths.^17^ However, most studies have failed to fully evaluate the economic impacts of heatwaves, such as by not considering the governmental subsidies.^38^ The intensity of heat waves and their adverse effects vary across different regions in China due to geographical variations, climatic characteristics, and economic development levels. Studies show that heatwaves more severely impact the eastern and southern provinces of China.^39^ Nevertheless, the adaptability brought about by economic development has increased the resilience of individuals with higher incomes to climate change.^40^ While most studies have focused on the short-term health impact of climate change, few have quantified the long-term economic impacts caused by heat waves in China.

### Climate and food system impacts

The intricate interplay between climate change, food, and disease presents a multifaceted challenge. Climate change wields a substantial influence on food systems, setting the stage for a cascade of health-related issues. Foremost, climate change exerts its influence on food production and security.^41^ For instance, the occurrence of extreme weather events poses a threat to crop yields, which threatens both the availability and diversity of our food supply.^42^ This, subsequently, can result in a higher prevalence of undernutrition, particularly in vulnerable populations in developing countries. In more developed economies, the confluence of elevated food prices and diminished food diversity can also contribute to a surge in chronic diseases linked to dietary factors. A notable example is the potentially reduced production and consumption of fresh fruits and vegetables, which can heighten the risk of cardiovascular diseases.^43^ Foodborne illnesses and food safety is also an important aspect.^44^ Rising temperatures and the increasing frequency of extreme weather events present substantial challenges to food preservation. Some foodborne bacterial pathogens, such as Salmonella and Listeria, thrive under these conditions, which may cause higher hazard of food borne illnesses.

## Climate change adaptation initiatives in China

### Adaptation initiative 1: carbon neutrality and health co-benefits

China’s NDC includes a commitment to peak its carbon emissions before 2030 and to achieve carbon neutrality by 2060, which means that the amount of carbon dioxide emitted is equal to the amount removed from the atmosphere.^5^ Given that China is the world’s largest greenhouse gas emitter, this commitment carries profound implications. While mitigation primarily targets the long-term goal of curtailing the root causes of climate change, mainly by reducing emissions, it is deeply entwined with adaptation. Reducing greenhouse gas emissions not only tackles the source of climate change but also lessens the severity of its impacts, enabling more manageable adaptation in the short term. Conversely, adaptation, which focuses on immediate responses to current or looming climate impacts, strives to diminish vulnerability to climate effects. In essence, effective mitigation creates a foundation for more successful adaptation, as both play complementary roles in China’s overarching strategy against climate change.

By 2030, China aims to increase the share of non-fossil fuels in primary energy consumption to around 25%, increase forest stock volume by 6 billion cubic meters compared to 2005 levels, and reduce carbon dioxide emissions per unit of GDP by more than 65% compared to 2005 levels. To effectively adapt to the effects of these NDC targets, the scientific and policy community must gain a comprehensive understanding of their short and long-term impacts on climate change. By assessing the potential outcomes of the NDC targets, we can identify areas that require additional resources or interventions and develop more effective adaptation strategies.^45^ As the world’s largest greenhouse gas emitter, China contributes approximately 28% of global emissions, a figure expected to rise until 2030, thereby intensifying climate change impacts. Given its large population, China’s per capita carbon emissions are lower than many developed nations.^46^

Reducing emissions in China not only aids in climate change mitigation but also offers significant co-benefits. Enhanced air quality results in health improvements, such as fewer cases of childhood asthma exacerbated by indoor air pollution.^47^ The drive towards cleaner energy promotes new industries and job opportunities while bolstering economic growth and energy security by lessening reliance on imported fossil fuels.^48^ However, to truly address climate-induced air pollution, a holistic approach, integrating both greenhouse gas reductions and robust adaptation measures, is imperative. Recent policies have indeed pivoted towards these goals, with a focus on industry regulations and the promotion of renewables.

### Adaptation initiative 2: improved early warning systems

**Fig. 4:**
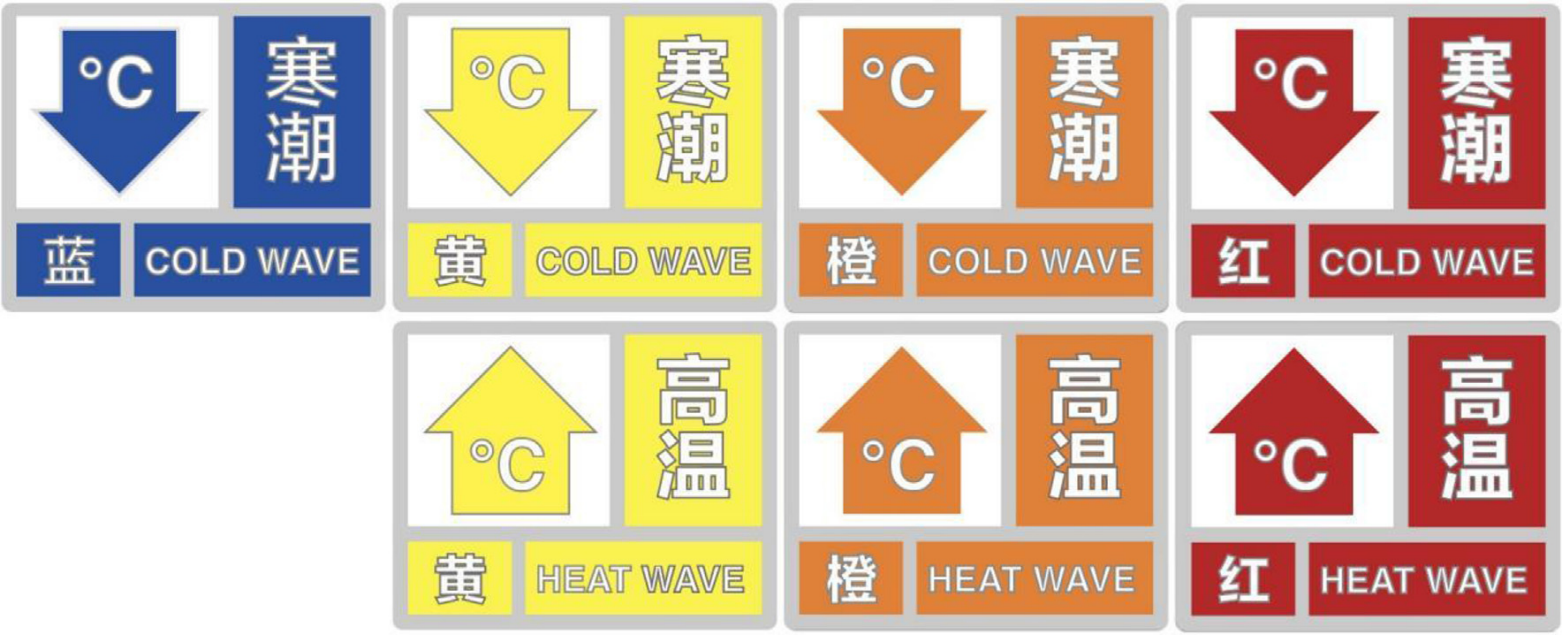
Heat health early warning system (HHEWS).

An early warning system is a critical component of disaster risk reduction, providing timely and accurate information about various extreme weather events. These events include heatwaves, cold spells, typhoons, heavy rainfall, severe thunderstorms, blizzards, strong winds, sandstorms, and more. Since 2000, some provincial meteorological bureaus in China have issued local weather warnings for these events. In 2008, the China Meteorological Administration (CMA) standardized this practice by introducing the “Regulations for the Issuance of Meteorological Disaster Warning Signals”, unifying 14 meteorological disaster warning signals across the nation in terms of their levels, symbols, and colors. The system is graded into four levels: Level I (particularly severe), Level II (severe), Level III (relatively severe), and Level IV (general), each indicated by colors red, orange, yellow, and blue respectively (details shown in Fig. 4).

### High-temperature early warning system in China

**Fig. 5:**
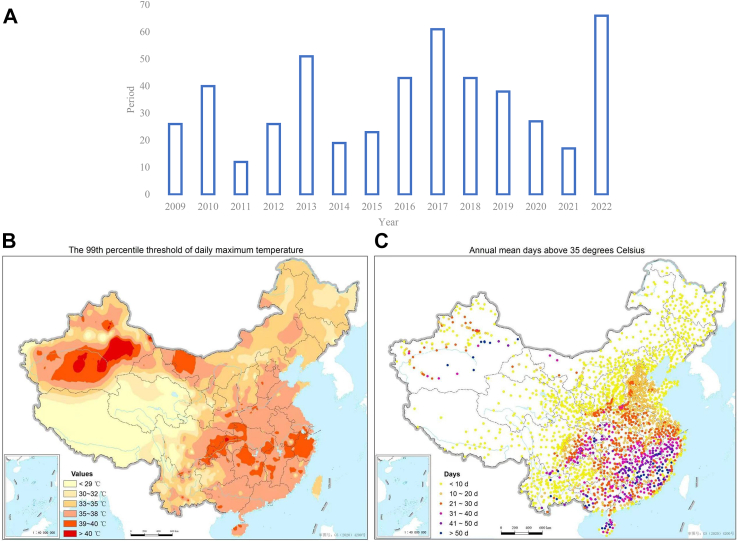
(A) Number of High Temperature and Heat Stroke Warnings Broadcasted by China Central Television. (B) Distribution of 99th percentile of daily maximum temperature in China over the past 10 years (2010–2020). Legend: Daily maximum temperatures surpassing the 99th percentile and their health implications. South of the Hu Line, these temperatures typically exceed 33 °C. Conversely, north of the Hu Line, they are predominantly below 33 °C. The northeastern and southwestern regions display temperatures between 33 and 35 °C, while the Qinghai-Tibet Plateau registers temperatures below 29 °C. (C) Annual average number of days with temperatures exceeding 35 °C in China over the past 10 years (2012–2022). Legend: In the last decade, most areas south of the Yangtze River experienced over 31 days with temperatures exceeding 35 °C annually. Regions witnessing more than 50 days of these high temperatures include Fujian, Jiangxi, Hunan, Chongqing, Xinjiang, among others. The Jianghuai region typically sees 20–30 days of such temperatures, while northern China regions usually have fewer than 20 days.

Specifically targeting heat-related events, national meteorological system alerts the public about the health risks posed by elevated temperatures. As depicted in Fig. 5a, China Central Television frequently broadcasts high-temperature and heat stroke warnings. In the Chinese meteorological context, any day recording a maximum temperature of 35 °C or above is labeled a high-temperature day. Fig. 5a charts the number of heat-stroke weather level forecasts presented on China Central Television’s Channel 1 (CCTV1) every summer from 2009 to 2022, factoring in biometeorological indexes that account for temperature and humidity. The system classifies heat stroke risk into three levels: Level III (likely to have heat stroke), Level II (prone to heat stroke), and Level I (very likely to have heat stroke). When at least five neighboring prefecture-level cities reach Heat-Stroke Level 2, a forecast warning is issued on CCTV1 for public consumption. A heatwave is defined as three or more consecutive days with temperatures exceeding 35 °C. Fig. 5b shows the distribution of 99th percentile of daily maximum temperature in China over the past 10 years (2010–2020), South of the Hu Line, these temperatures typically exceed 33 °C. Conversely, north of the Hu Line, they are predominantly below 33 °C. The northeastern and southwestern regions display temperatures between 33 and 35 °C, while the Qinghai-Tibet Plateau registers temperatures below 29 °C. Fig. 5c shows annual average number of days with temperatures exceeding 35 °C in China over the past 10 years (2012–2022). In the last decade, most areas south of the Yangtze River experienced over 31 days with temperatures exceeding 35 °C annually. Regions witnessing more than 50 days of these high temperatures include Fujian, Jiangxi, Hunan, Chongqing, Xinjiang, among others. The Jianghuai region typically sees 20–30 days of such temperatures, while northern China regions usually have fewer than 20 days. Current protective recommendations include staying indoors during peak heat hours, wearing sunscreen and protective clothing, limiting outdoor work, and implementing cooling measures. In 2022, a surge in high-temperature days led to many record-breaking temperatures and a consequent spike in warning broadcasts, including an unprecedented red-level warning. However, some experts raise concerns over the lack of a universally applicable heatwave definition, considering the varied climatic conditions and adaptive capacities across different regions. A study in Shanghai found that such early warning systems, particularly those targeting heatwaves have been effective in reducing mortality during such events.^49^

### Adaptation initiative 3: cities and spatial planning as protection against extreme weather events

The United Nations Environment Programme has noted that “Cities are a key contributor to climate change”.^50^ In 2017, China launched a pilot program for the construction of climate-resilient cities in 28 regions and introduced action plans for urban adaptation to climate change.^51^ Urban spatial planning measures have been proposed for extreme weather and climate events such as heavy precipitation, high temperature, drought, typhoon, freezing, and haze, including optimizing urban infrastructure, renewing old residential areas, and enhancing the function of green spaces, forests, lakes, wetlands, and ecosystems to conserve water sources, regulate temperature, and maintain water and soil. Box 3 shows China’s macro spatial planning policies for climate change adaptation.^52^, ^53^, ^54^, ^55^, ^56^, ^57^, ^58^ In 2019, the National Standard of the People’s Republic of China “Technical for climatic feasibility demonstration in master planning” was released,^59^ suggested that urban planning should adapt to climate change by using spatial indicators, such as urban form, green space layout, green space rate, and functional zoning. In 2023, eight Chinese government ministries jointly released a notice on deepening the pilot construction of climate-resilient cities, proposing to actively explore the paths and models of climate-resilient city construction and effectively improve the city’s ability to adapt to climate change.^60^ Over the past 15 years, China has also developed urban construction strategies such as low-carbon cities and sponge cities to secure the resilience of cities to climate change and promote the well-being of the public.^61^^,^^62^ Effective urban planning, including proper building layouts, urban sky corridors, and vertical greenery, enhances air circulation, reduces pollutants, and mitigates the heat island effect, thus improving the local microclimate. Such measures not only help decrease mortality from high temperatures but also benefit residents in high-density areas by potentially reducing infectious diseases.^52^, ^53^, ^54^

Box 3 **China’s macro spatial planning policies for climate change adaptation**

**Table:** 

1. Climate change integration in urban planning:	Technical Specification for Climate Feasibility Demonstration of Urban Master Planning (issued by the China Meteorological Administration in 2014) emphasizes climate change adaptation in urban planning through spatial indicators such as urban form, green space layout, green space ratio, and functional zoning. National Land Planning Outline (2016–2030) proposes measures to address climate change, including strengthening ecosystem protection, promoting green and low-carbon city development, and transforming agricultural production methods.
2. A series of urban construction strategies (low-carbon cities, sponge cities, resilient cities):	Low-carbon cities: Since 2010, China has identified six low-carbon pilot provinces and regions and 81 low-carbon pilot cities. Each mainland province has at least one low-carbon pilot city, except for some western regions (Ningxia, Tibet, and Qinghai). Sponge cities: From 2015 to 2016, China identified 30 pilot sponge cities across different regions, scales, and types, aiming to enhance rainwater absorption, storage, infiltration, purification, and utilization, while reducing urban flooding and improving water environment and ecological quality. Resilient cities: The “Proposal of the Central Committee of the Communist Party of China on the 14th Five-Year Plan for National Economic and Social Development and the 2035 Visionary Goals” (2020) introduced the concept of constructing “resilient cities” to enhance cities’ resilience to disasters.
Specific Measures for Policy-based Spatial Planning–Chinese Practice Examples and Health Effects:	
1. Building layout, urban sky corridors, and vertical greening:	Examples: Hong Kong 2030+: Planning Vision and Strategies for Moving Beyond 2030, Guangzhou “Cool City” Initiative. Health effects: Research in Hong Kong suggests that urban planning measures to mitigate the heat island effect and improve the living environment can help reduce mortality from high temperatures. Additionally, residents in high-density residential areas may face higher risks of tuberculosis.
2. Public/slow transport system construction:	Examples: “Beijing 14th Five-Year Plan for Transportation Development and Construction” and “Shanghai Street Design Guidelines.” Health effects: Improvements in China’s transportation system can increase opportunities for physical activity, control weight and blood pressure, prevent chronic diseases, reduce stress and anxiety, improve mental health, and decrease air pollution. Example: Wuhan’s East Lake Greenway, the largest urban greenway in China, providing activity space, improving water quality, regulating microclimate, and optimizing the ecological environment. Health effects: Natural experiments on the East Lake Greenway demonstrate the positive impact of urban green spaces on the physiological and psychological health of residents.
3. Adequate and high-quality blue-green open space	Practical example: Wuhan’s East Lake Greenway (the largest urban greenway in China)–provides activity space, improves water quality, regulates microclimate, and optimizes ecological environment. Health effects: Natural experiments on the East Lake Greenway have shown that urban green spaces have a positive impact on the physiological and psychological health of residents.^6^
4. Planning of disaster prevention infrastructure, emergency shelters, and medical and health service facilities.	Examples: Beijing Urban Master Plan 2016–2035, Chongqing Urban Infrastructure Construction “14th Five-Year Plan,” and the 14th Five-Year Plan for Urban Infrastructure Development in Guangzhou. Xiamen Haicang’s sponge city practice during Typhoon Moranti in 2016. Health effects: Proper layout of medical facilities and emergency shelter planning effectively reduces disaster mortality and controls the spread of infectious diseases.

However, the effectiveness of the measures implemented in these pilot cities remains to be evaluated. For instance, one perspective posits that Chinese sponge cities, by integrating blue-green systems with traditional grey infrastructure, represent a paradigm shift in sustainable water resource management. They are effective for flood risk reduction; however, they are unlikely to be a panacea for flooding problems in cities as they cannot deal with low-probability and high-damage events.^63^ The evident technological disparities and constraints between developing and developed countries must be addressed.^64^ Factors such as the unavailability and uncertainty of rain garden systems, green roof systems, tree planters, infiltration planter systems, and underground infiltration and monitoring systems can pose significant challenges to the effective implementation of adaptive measures.^64^^,^^65^ It’s also vital to recognize the spatiotemporal differences and thresholds of each measure. For example, data from 162 cities in China suggests a nonlinear impact of urban and industrial structures on surface urban heat island. As the temperature rises, adaptive measures may become ineffective, or even produce negative effects.^66^

Improvements in the transportation system in China can increase opportunities for daily physical activity by encouraging daily activities such as walking or cycling, helping to control weight and blood pressure, preventing obesity and chronic diseases, relieving stress and anxiety, improve mental health, while also mitigate air pollution from exhaust fumes.^55^ Many cities have emphasized the development of public transportation and non-motorized traffic systems in planning strategies such as the “14th Five-Year Plan for Transportation Development and Construction in Beijing” and the “Shanghai Street Design Guidelines”.^67^^,^^68^ After implementing a series of green public transportation initiatives, Nanning city saw its green travel mode share exceed 80% in 2021.^69^ Additionally, green infrastructure and open blue-green spaces can improve air quality and encourage daily physical activities. For example, the construction of the East Lake Greenway in Wuhan, the longest urban greenway in China, has improved water quality and the ecological environment of East Lake, and a local natural experiment showed that it had a positive impact on the physical and mental health of surrounding residents.^56^^,^^57^ It’s worth noting that the ability of these transportation and green infrastructure initiatives to mitigate the effects of climate change might be constrained under scenarios of rapid global warming. For instance, outdoor activities or travel can be hazardous under extreme high temperatures. A Chinese empirical study has pointed out that the green view index shows a stronger cooling effect than that of green space area under extreme heat.^70^

China has effectively managed the impacts of climate change, including floods, heavy rainfall, droughts, and water supply issues, by improving infrastructure like flood control zones, urban pipe corridors, and lifeline systems, thereby safeguarding public health and safety. Cities such as Shanghai, Xi’an, and Chengde have implemented emergency shelter plans to protect residents further.^71^, ^72^, ^73^ In August 2023, China will roll out the “Urban and Rural Public Health Emergency Space Planning” initiative, designed to enhance the city’s resilience to natural disasters and potential climate change-induced infectious diseases.^51^ This initiative strategically allocates land, space, and facilities for peace times and public health emergencies. Evidence suggests that China’s well-organized medical facilities and emergency shelters have proven effective in mitigating disaster-related mortalities and curbing the spread of infectious diseases during significant epidemics.^58^ However, as climatic events become more sudden, frequent and severe, it’s essential to continuously reassess and upgrade these measures in line with the latest climate warning and modeling systems. For instance, in July 2023, due to the combined effects of typhoons and topography, Beijing and its surrounding areas in China experienced a catastrophic heavy rainfall event. Although the government promptly initiated flood prevention emergency responses and took corresponding measures,^74^ there were still casualties and property losses.^75^ Under severe climate change, the applicability and effectiveness of spatial planning policies and measures need to be tested in a timely manner.

### Adaptation initiative 4: optimize indoor environments

Indoor environments can protect against extreme weather events by providing a safe and comfortable space for people to shelter from the extreme weather, ranging from non-optimal temperature, air pollution from landscape or wildfire, and natural and man-made disasters. Indoor environments are essential for adaptation by providing a safe and secure shelter during extreme weather events, indoor environments can contribute to climate change adaptation and improve public health and safety. During heatwaves, air conditioning indoors offers temporary relief and reduces heat-related illness risks. In extreme cold, indoor heating prevents hypothermia and related risks. Buildings with strong construction and features like insulation and weather sealing stay cool on hot days and warm on cold ones. They also resist extreme events like hurricanes and floods. According to the 2022 Lancet Countdown report on health and climate change in China, air conditioning units per 100 households increased from 18.3 in 2000 to 117.7 in 2020. These units potentially prevented around 23,300 heatwave-related deaths in 2020, with 77% being individuals aged 65 or older. However, their usage also led to around 300 million tons of CO^2^ emissions, six times the amount in 2000.^76^

Adaptation to climate change in indoor environments can be achieved by reducing carbon emissions, which is also linked to mitigation efforts. As climate change progresses, more people are expected to spend more time indoors, necessitating enhancing indoor environments to protect against health hazards. Enhancing indoor environments can be achieved by reducing indoor air pollution and improving thermal comfort, reducing energy consumption. Climate change is likely to have a significant impact on indoor environmental quality (IEQ), particularly indoor temperature and humidity. In China, improving the energy efficiency of buildings can significantly reduce greenhouse gas emissions, lower energy consumption, improve IEQ, and reduce health risks associated with indoor pollution. The Chinese government has introduced policies to promote energy-efficient buildings and IEQ, such as the “Green Building Evaluation Standards” and the “Ten Cities, Thousand Villages” projects, among others.^77^

In addition, as indoor air quality is significantly influenced by outdoor air quality, climate change possesses an inevitable impact on both indoor and outdoor air quality. For example, a wildfire produces a large amount of fine particulate matter, which will worsen ambient air pollution and then enter the indoor environment via ventilation or even infiltrate the indoor environment via air infiltration when windows close.^78^ Use of an air purifier may be an effective adaption measure.^79^^,^^80^ However, owing to difference in the socioeconomic resources, weather conditions, and air quality in different regions of China, the health benefits and cost-effectiveness of using air purifiers to remove indoor fine particulate matter are very different.^81^^,^^82^ Modeling studies show that in Chinese cities where the ratio of average annual outdoor PM_2.5_ concentration to GDP per capita is low, greater net benefits are derived from using air purifiers to achieve lower indoor PM_2.5_ targets. Specifically, 89 cities with the lowest such ratios reaped the most benefits when air purifiers reduced indoor PM_2.5_ levels to the WHO AQG 2021 standard of 5 μg/m3. Conversely, 12 cities with high outdoor PM_2.5_ but low GDP per capita maximized net benefits at a less stringent indoor target of 35 μg/m^3^. Remarkably, in 70 largely underdeveloped cities, using air purifiers was deemed not cost-effective even at this relaxed target.^81^ This underscores the need to critically assess the equity implications of relying on air purifiers as an adaptation to climate change. Addressing this disparity necessitates advancing sustainable economic growth and prioritizing air quality, especially in cities that are economically lagging yet highly polluted.^82^

### Adaptation initiative 5: natural vegetation, green space, and cooling infrastructure

China has focused its efforts on adaptation measures in cities. Pilot programs for eco-cities and sponge cities are initiatives to incorporate strategies such as green roofs and walls, modifications to building form and layout, and the use of heat-resistant construction materials to improve energy efficiency, reduce the urban heat island effect, and manage stormwater runoff. Increasing vegetative cover in urban areas, such as dividers in busy roads, around community centers, can also have multiple benefits, including lowering outdoor temperatures, reducing demand for building cooling, and sequestering carbon while managing run-off and pollution. This is coupled with designs for houses and buildings that can withstand flooding, such as amphibious houses that can float on the water during floods.^83^ Lacking is before and after studies on built environment changes and community health outcomes.

**Fig. 6:**
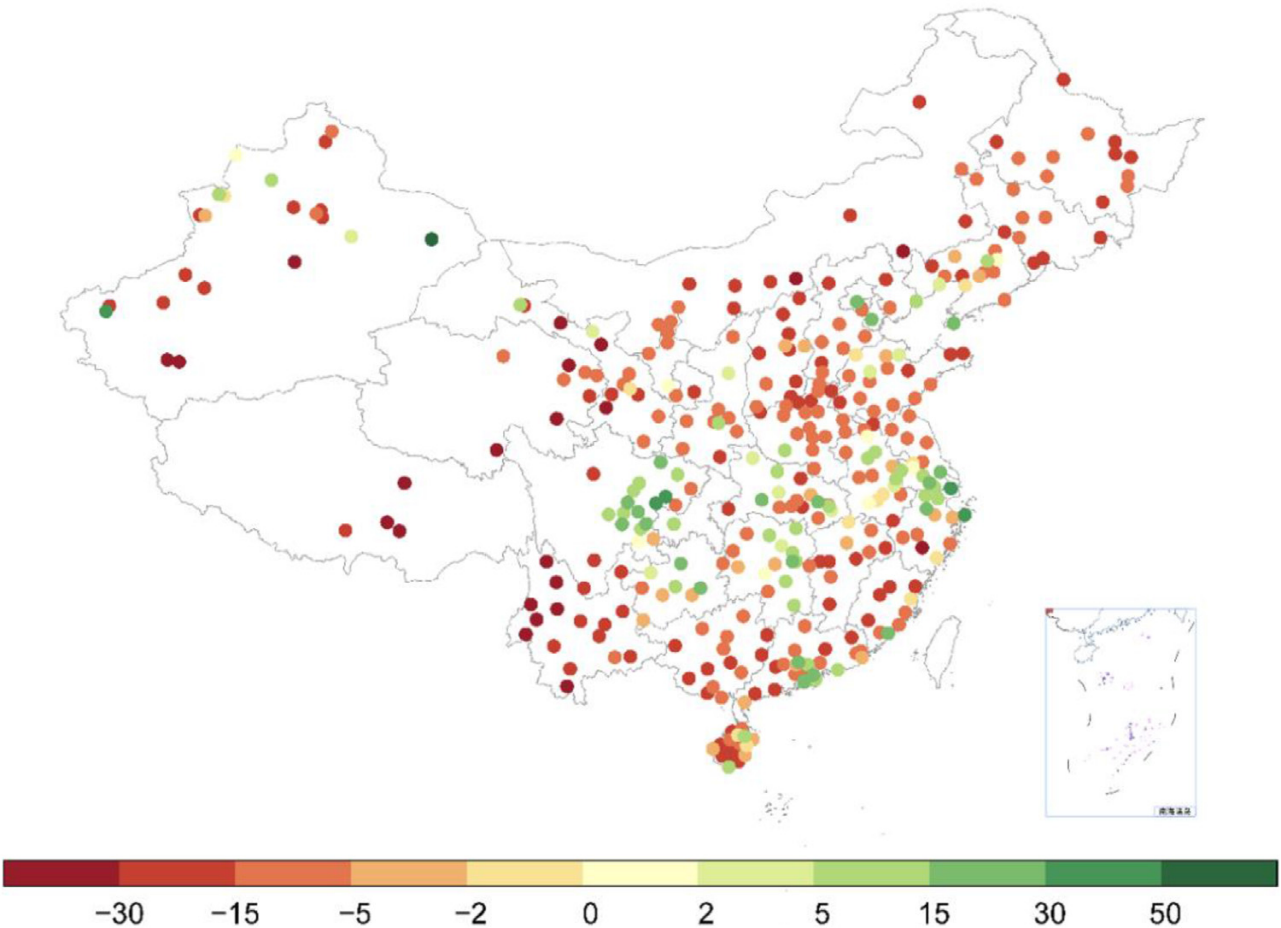
The percentage change (%) of NDVI for 361 cities in China Mainland from 1990 to 2020. Data source.^8^

Green spaces, as defined by WHO, are lands with any vegetation cover (e.g., grassland, forests). Growing evidence shows that greenspaces can play a crucial role in climate change adaptation by providing multiple benefits to the environment, society, and human health. However, there has yet to been country-level guidance, probably owing to a lack of health studies. Global evidence indicate green spaces could improve the microclimate by preserving biodiversity^84^ and reducing the urban heat island effect,^85^ carbon emissions,^86^ air pollution,^87^ noise,^88^ and the risks of certain natural disasters like flood and mudflow.^89^ For society, greenspaces can improve social interaction and the physical level of the public.^90^ A series of studies from China have assessed the associations of greenspace with various health outcomes from different aspects of greenspace, including greater greenness was associated with lower mortality,^91^^,^^92^ lower metabolic syndrome,^93^ lower likelihood of frailty,^94^ lower cognitive impairment,^95^ and decreased odds of asthma in children.^96^ However, although greenspace was commonly associated with these benefits, few greenspace-health studies simultaneously accounted for these factors. Fig. 6 shows the percentage change (%) of NDVI for 361 cities in China Mainland from 1990 to 2020.^97^

From 1985 to 2019, the overall area of grassland in China decreased by −3.29%, and the forest area increased by 4.34%.^98^ However, despite the government’s effort to increase green space coverage, China still faces a decline in population-weighted green space exposure level due to the unprecedented rate of urbanization and industrialization.^99^^,^^100^ While forest stock increased on an ecological scale in China, urban green space may not have benefited because of the peri-urbanization.^101^ However, there is uncertain evidence of whether green space may experience an environmental Kuznets curve (EKC) in urban areas as per capita income increases and urban living becomes in demand.^102^ EKC is a hypothesis that postulates an inverted-U-shaped relationship where environmental pollution becomes higher over the course of development and decreases once per capita income achieves a level. Evidence on the health effects of green spaces from China, especially on different types of green spaces, health outcomes, and the pathway analysis, would be valuable to understand the health implications of greenspace better and inform public health policies and urban planning decisions to enhance the community’s resilience to the impacts of climate change. Currently, public green space, such as parks surrounding residential blocks, is still relatively limited and unequally distributed within and across cities in China. The creation and preservation of accessible greenspaces should still be prioritized, especially for densely populated blocks and cities. Additionally, more scientific evidence and community engagement could be involved in the planning and designing of green spaces, which can help maximize the cost benefits. Supplementary Boxes S2 and S3 show two case studies on climate change adaptation and health Implications in Shenzhen.

### Adaptation initiative 6: raise public awareness and protecting vulnerable populations from climate-related risks

Identifying populations and locations at high risk of heat vulnerability is crucial for effective interventions. Certain population groups show different vulnerabilities to temperature impacts. Evidence is emerging. A study in Hefei, China, found that girls and preschool children were more susceptible to the combined effect of temperature and air quality index (AQI), while AQI and humidity more influenced boys and school-age children.^103^ Another study revealed that school-age children in the warm season and various subgroups, except preschool children in the cold season, were susceptible to temperature variability (TV).^104^ Elderly individuals, residents of poor communities, and those with chronic diseases are also more vulnerable to extreme temperature events.^105^^,^^106^ For pregnant women and fetus, exposure to significant heat events can heighten the risk of preterm birth (PTB), especially when the thermal event surpasses specific intensity and duration thresholds, notably in the first four gestational weeks and between week 21 and the last month.^107^

Bottom-up citizen-driven adaptation efforts are essential to protection. Being aware of climate change is frequently seen to be crucial for gaining public support for programs of mitigation and adaptation.^108^^,^^109^ In the past ten years, China has experienced a reasonably high level of public knowledge of climate change, and most people acknowledge that the cause is human activity, which is in line with the scientific community.^110^ Another recent study concluded that the general attitude towards climate change is evolving in a favorable direction as public understanding of it rises.^111^ It is also important to note that Chinese respondents were more concerned about climate change than terrorism, economic progress, or education.^112^ They also believed that conservation is more crucial than climate change but less urgent than air pollution.^112^

In China, climate change awareness varies among different populations.^113^ Some studies also looked at whether sociodemographic, psychological, and cultural factors, like age, gender, rural versus urban status, and income, can most accurately predict how the public perceives and reacts to climate change.^112^ A study undertaken in Suzhou, Hefei, and Xiamen on the risk perception of the elderly Chinese showed that the respondents were highly aware of climate change, and they were very worried about the health impacts of climate change.^114^^,^^115^ Research conducted between 2017 and 2019 in six Chinese cities: Dongguan, Guyuan, Hangzhou, Yancheng, Yangzhou, and Suzhou, indicates that residents in heavily polluted areas often conflate air pollution with climate change. However, individuals with higher education, greater wealth, and younger age demonstrate a better understanding of the scientific nuances of climate change and its impacts.^116^ The study found that women and the elderly were more concerned about adaptability to climate change and related health issues.^116^ Another survey assessed public awareness, risk perceptions, policy preferences, and behaviors concerning climate change. A significant majority reported having some knowledge of climate change. Of these respondents, a fraction considered themselves “very knowledgeable,” while a small percentage had never heard of it.^117^

Other research, some respondents were willing to take personal action to combat climate change, and they paid more attention to climate change or agreed that it harms individuals and society.^118^ However, they also tended to have faith in the government to address the issue or think that fiscal and taxation policies are the most effective policy measures to combat climate change.^118^ Other advantages of increasing carbon pricing include improvements to air quality and health.^119^ For instance, China’s implementation of a coal tax might prevent the loss of roughly 3 million lives by 2030. Revenue from carbon taxes can be channeled into priority sectors such as health, education, infrastructure, and green investments, as well as compensating those adversely affected. Moreover, improved environmental conditions can enhance public health, reducing mortality rates^119^ In general, awareness and concern about climate change among the Chinese population are growing, with many acknowledging its detrimental impacts.^112^

### Adaptation initiative 7: environmental engineering technologies for health

Advancing environmental technologies and safeguarding public health are interconnected. Especially, the future pollution scenarios are expected to differ much from the current situation, arising from both the climate change and its adaptation measures. Therefore, it’s pressing to develop proactive environmental technologies against the predicted future pollution conditions. In view of this, China released a special plan for scientific and technological innovation in the field of ecological environment as part of the “14th Five-Year Plan” in 2022. This national framework sets the direction for future developments in environmental engineering technologies. Notably, to effectively address the challenges posed by climate change and adaptation measures, the plan recognized the critical need for technological innovations in the following key areas.

First, climate change has presented new challenges in the prevention and control of pollution by influencing pollution emissions and weather conditions.^120^, ^121^, ^122^, ^123^ Among the pollutants influenced by climate change, the ozone issue stands out as the most significant; known as the ozone climate penalty, referring to the phenomenon where climate change influences the formation and distribution of ozone in the atmosphere, leading to increased levels of ground-level ozone.^124^ Moreover, as significant efforts have been made in controlling PM_2.5_ and implementing carbon neutrality measures, the concentration of PM_2.5_ in China has been and is expected to continue decreasing.^125^ However, it is important to note that without additional measures, this reduction in PM_2.5_ concentration is likely to exacerbate the ozone pollution situation.^126^^,^^127^ Therefore, special attention has been given to the synergistic control of PM_2.5_ and ozone.^128^^,^^129^ These efforts include the development of more sensitive and portable instruments for the three-dimensional detection of air pollutants, the observation-modeling coupled study to understand better the PM_2.5_–ozone coupling mechanism and the nonlinear relationship with precursor emissions, and an improved model to assess better the impact of climate change, among other factors.

Second, the impact of climate change on emerging pollutants like persistent organic pollutants (POPs) is of growing concern.^130^, ^131^, ^132^ Despite the advances in these years, substantial technological innovations are underway.^133^ There is an immediate need for instruments that enable rapid screening, monitoring, and early warning, but such tools are currently unavailable. To effectively prevent and control pollution, a comprehensive understanding of the sources of these pollutants, their transport and transfer processes across various environmental media, and their toxicity mechanisms is essential. Furthermore, developing more efficient treatment technologies and green substitution solutions is crucial.

Finally, it is important to note that while novel technologies for climate adaptation are necessary, their potential environmental and health impacts are not yet fully understood and require further assessment.^133^, ^134^, ^135^ For instance, the widespread utilization of solar and wind energy can result in a significant amount of new industrial solid waste, such as discarded solar panels, which currently lack effective recycling methods.^136^ Incineration for power generation is considered one of the effective ways to treat solid waste,^137^ but this process will generate highly toxic air pollutants and may emit even more greenhouse gases.^138^^,^^139^ Consequently, China is actively developing waste-recycling technologies and implementing improved strategies for assessing the environmental and health impacts of potential novel solutions on a life-cycle basis.

### Adaptation initiative 8: government academic partnership in science, technology, and policy

China needs to assume that the increase in extreme temperatures and threat to human health will continue in the near future, and take adaptation actions over the next few decades. Developing effective communication channels between government agencies, research organizations, non-governmental organizations, and the general public will lead to synergy for continued national adaptation strategy, with regional experiences and know-how. It is essential for scientists and policymakers to work collaboratively to answer specific research questions oriented toward the development of adaptation policies. Addressing climate-related health issues requires a coordinated effort across all government departments, and “health for all” should be integrated into decision-making processes in environmental, urban planning, energy, transport, and public health.

The “National Climate Change Adaptation Strategy 2035” launched by governmental entities is a crucial step towards successful climate change adaptation. Currently, the guideline includes a key component of public health and disaster emergency response. The implementation at localities will likely evolve to minimize its adverse effects on human health. Not all adaptation measures will be suitable in all regions, but positive steps taken in these innovative strategies, such as using green infrastructure to mitigate urban heat island effects, can also help reduce heat-related morbidity and mortality. Additionally, integrating climate change adaptation into existing health policies and programs can facilitate a more coordinated and effective response to climate-related health risks in China.

The scientific community must advance the science of temperature-related adaptation strategies/measures and engage the government and the public. Transdisciplinary and cross-sectoral collaboration is required to reduce uncertainties in decision-making and evaluate the effectiveness of public health adaptation strategies. Funders need to increase their investment in transdisciplinary and collaborative research to understand better the health risks of exposure to heat/cold and related climate action priorities.

A culture of innovation benefits public health through technological solutions for adaptation to climate change. New technologies are continually emerging. For instance, while air conditioning offers protection against heatwaves, transitioning to systems that utilize cooling towers or district distribution can be more energy-efficient and decrease heat emissions.^140^ Surface reflectivity in urban areas is one of the most important determinants of the magnitude of the heat island effect. Increasing reflectivity, for example, by painting light-colored surfaces, can reduce urban air temperatures by 1–3.5 °C.^141^ One study found that in combination, increasing the albedo and the vegetated area can lead to a 48% reduction in annual emergency service calls and offset 40–99% of the projected increase in heat-related mortality arising from climate change.^142^^,^^143^ Increasingly, big data and internet-of-things tools can inform decision-makers in real-time to make more effective decisions on resource needs and flow in cities. Sensors and automated or unmanned systems (e.g., on-demand watering systems) are increasingly common under smart-cities frameworks to save, recycle, and upcycle water before or during droughts and floods.^144^^,^^145^ Although technology-driven solutions are well studied, and new technologies are continually developed, efforts are needed to understand barriers in localities for many cities without the resources to access, implement and maintain them.

## Conclusion

China’s path toward successful climate change adaptation is crucial to safeguarding the remarkable health gains achieved over the past five decades. As the country faces the challenge of climate change, tailored strategies are necessary to multifaceted challenges and preserve public health achievements. China’s commitment to achieving carbon neutrality by 2060 can tackle the root causes of climate change and also offers co-benefits for health.

Adaptation efforts need to address regional disparities and surmount the constraints of top-down approaches to climate change management. Early warning systems play an essential role in preparing for extreme weather events. However, a basic color-coding system may not fully represent the diverse severity of events across various regions or account for region-specific adaptation measures. Adopting a percentile system for the local heat index or extreme weather events could better align with region-specific adaptation strategies. As urbanization progresses, there’s an intrinsic connection between urban morphology and the indoor environment. Spatial planning in cities is fundamental for cultivating resilient urban structures and environments. Integrating natural vegetation and green spaces into urban planning not only offers multiple benefits but also plays a role in mitigating the urban heat island effect and promoting carbon sequestration. To further enhance climate change adaptation, it is crucial to ensure the effective integration of these urban and indoor strategies. Specifically, optimizing indoor environments strengthens China’s resilience against climate change by offering refuge during extreme weather events. All these efforts should be evaluated within the broader framework of holistic urban development.

To address these challenges, adaptation strategies must encompass green city designs, improved indoor environments, robust meterological early warning alert systems, and a co-benefits approach intertwining human health with carbon neutrality and technological advancements. Emerging technologies, such as cooling towers and district distribution systems, alongside tools like increasing surface reflectivity, offer sustainable solutions against urban heat effects. Integration of big data, sensors, and the internet-of-things is paramount for effective climate change management. Understanding barriers to technological adoption, especially in resource-limited areas, is crucial. As China innovates and strengthens its climate adaptation measures, it simultaneously promotes global collaboration, positioning climate resilience as an international imperative. Future initiatives should deeply explore the interplay between technology, urbanization, climate variations, and public health. By emphasizing sustainable infrastructure, renewable energy, and global collaboration, China not only fortifies its own resilience but also paves the way for unified global efforts.

## Author contribution

John S. Ji led the study with contributions in conceptualization, data curation, funding acquisition, investigation, methodology, project administration, resources, supervision, validation, visualization, writing of the original draft, and review & editing. Yanjie Xia was responsible for conceptualization, data curation, investigation, methodology, validation, visualization, and both the original and review writing phases. Linxin Liu contributed to review & editing of the figures and visualization, and validation of main findings. Specific thematic contributions include: Weiju Zhou specialized in the sections concerning floods and the Beijing case study; Renjie Chen and Haidong Kan on air pollution; Qinghua Hu on water topics and infectious disease; Jingkun Jiang and Zheng Guangjie on environmental technology; Tiantian Li on heatwaves; Yi Li and Yanxiang Liu on the meteorological early warning system; Ying Long and Yue Ma on urban design; Yuebin Lv, Xiaoming Shi, and Qiyong Liu in conceptualization and validation; Jian Ma and Lei Xu on the One Health and disease transmission patterns; Pelin Kinay on qualitative research on climate change; Shilu Tong on conceptualization of key ideas and editing; Yang Xie on the economic impact of climate change; Changzheng Yuan on food and climate relations; Huateng Zeng on the Shenzhen case study; and Bin Zhao on the optimal indoor environment. Wannian Liang, Margaret Chan, and Cunrui Huang contributed in methodology and resources. All authors reviewed, edited, and gave final approval for the manuscript.

## Editor note

The Lancet Group takes a neutral position with respect to territorial claims in published maps and institutional affiliations.

## Declaration of interests

Authors declare no competing interests.

## Appendix A Supplementary data

Supplementary Web text S1 and Boxes S1–S3
