# Supplementary material for: China’s public health initiatives for climate change adaptation
Source: Lancet Reg Health West Pac. 2023 Nov 15;40:100965. doi: 10.1016/j.lanwpc.2023.100965 (PMC10730322; doi:10.1016/j.lanwpc.2023.100965)
Supplement: Supplementary Web text S1 and Boxes S1–S3 [file mmc1.docx]

**Supplementary Appendix: China's public health response to climate change: climate risks and adaptation initiatives**

[Web text 1. The cumulative incidence of 3 infectious diseases in China at county scale (100000 persons) 1](#_Toc579958084)

[Box S1. Case Study: 2023 Typhoon Doksuri 5](#_Toc2074727702)

[Box S2. Case Study: Climate Change Adaptation and Health Implications in Shenzhen's Urban Development 6](#_Toc1256511175)

[Box S3. Case Study: Shenzhen's dedication to eco-friendly and health-centric urban development 9](#_Toc1976617274)

# Web text 1. The cumulative incidence of 3 infectious diseases in China at county scale (100000 persons)

Evidence shows that infectious diseases are prone to be impacted by climate change.^1^ Climatic conditions are becoming increasingly suitable for the transmission of multiple infectious diseases, by directly affecting biological characteristics of pathogens (eg, growth, survival, and virulence) and their vectors, and by indirectly favoring transmission through the modification of ecosystems and changes in human behaviour.^2^ Precipitation affects mosquito densities by affecting surface water, which in turn affects mosquito breeding sites.^3^ In Karamay, Xinjiang, although it is in an arid region, places with surface water (like marshes) can become breeding grounds for mosquitoes and there is still a risk of dengue transmission (Location 1). Temperature affects dengue transmission by influencing mosquito behavior, external incubation period, and transmission probability.^4^ Chongqing, one of the hottest cities in China, has an increased risk of dengue in the future (Location 2). Typhoon is associated with increased risk of dengue in south China.^5^ As typhoons become more frequent,^6^ Hainan, one of the most typhoon-affected regions, may re-emerge as a dengue-outbreak region (Location 3). Xishuangbanna has a very high risk of imported cases due to its border location (Location 4).^7^ High population density and mobility also lead to a higher risk of disease transmission.^8–10^ A total of 1,153 cases reported during the 2017 outbreak in Hangzhou (Location 5). The epidemic in Guangzhou in 2014 was made possible by the combination of favorable conditions for all factors,^11^ such as imported cases, mosquito density, climate conditions, population density and human mobility, with 36,851 cases in 2014 (Location 6). The prevalence of plague is linked to climate, vegetation and the density of rodents and fleas.^12^ Climate conditions (temperature, precipitation) indirectly affect rodent density, linked to plague prevalence.^12^ Increased precipitation favors rodents in northern arid areas, while high precipitation negatively affects southern humid areas (Location 7). Drought-related degradation of grasslands in Inner Mongolia has been speculated as the main cause of the plague cases reported in Beijing in November 2019, including two pneumonic plague cases (Location 8).^13,14^ Negative association found between vegetation and rodent density in Siziwang Banner (Location 9). ENSO influences rodent and human plagues through precipitation and temperature variations in China.^15^ El Nino years facilitate rodent outbreaks and subsequent human plague occurrences (Location 10).^15^ Highly vulnerable to climate variations and severe animal plague area.^16^ Plague risk increasingly sensitive to climate change in the Tibetan Plateau (Location 11).^16^

ENSO-driven climate variations expand vibrio cholerae to higher latitudes and prolong epidemics. Sea-level rise increases inland water salinity, expanding vibrio cholerae habitat. Floods and droughts increase water source contamination and human exposure.^17^ As a result, the risk of cholera outbreaks in the coastal cities is increasing (Location 12). The hot and humid environment of Sichuan was conducive to the propagation of vibrio cholerae, leading to cholera outbreaks in the Republic of China (Location 13).^18^ The lack of adequate water facilities and services leads to increased illness from diseases such as cholera.^19^ In Xinjiang, water scarcity due to drought requires centralized management and redistribution of drinking water, increasing the risk of cholera outbreaks when water sources are contaminated (Location 14).

In summary, the intricate links between humans, animals, and their ecological environments are apparent in light of complicated climate changes under the One health conception.^20^ Changes in temperature and precipitation brought about by climate change, as well as the occurrence of extreme weather events, affect the risk of infectious disease outbreaks. Dengue outbreaks in recent years have been associated with increasingly frequent human movements and increased population densities, the spatial heterogeneity of climate-driven plague due to the different geographical distributions of rodents, and cholera outbreaks are affected by the contamination of water resources due to flooding. In the future, we should be vigilant about the impact of climate change on human health. Improve understanding of ecology, entomology, zoology and epidemiology, continue to enhance vector surveillance and control, strengthen environmental protection and governance, and develop locally targeted response strategies.

Supplemental reference

1 Flahault A, de Castaneda RR, Bolon I. Climate change and infectious diseases. Public Health Reviews 2016; 37: 21.

2 Microbe TL. Climate change: fires, floods, and infectious diseases. The Lancet Microbe 2021; 2: e415.

3 Tian H, Huang S, Zhou S, et al. Surface water areas significantly impacted 2014 dengue outbreaks in Guangzhou, China. Environmental Research 2016; 150: 299–305.

4 Watts DM, Burke DS, Harrison BA, Whitmire RE, Nisalak A. Effect of Temperature on the Vector Efficiency of Aedes aegypti for Dengue 2 Virus. The American Journal of Tropical Medicine and Hygiene 1987; 36: 143–52.

5 Li C, Zhao Q, Zhao Z, Liu Q, Ma W. The association between tropical cyclones and dengue fever in the Pearl River Delta, China during 2013-2018: A time-stratified case-crossover study Tropical cyclones and dengue fever. Plos Neglect Trop Dis 2021; 15: e0009776.

6 Cai W, Zhang C, Suen HP, et al. The 2020 China report of the Lancet Countdown on health and climate change. The Lancet Public Health 2021; 6: e64–81.

7 Yue Y, Liu X, Xu M, Ren D, Liu Q. Epidemiological dynamics of dengue fever in mainland China, 2014–2018. International Journal of Infectious Diseases 2019; 86: 82–93.

8 Wesolowski A, Qureshi T, Boni MF, et al. Impact of human mobility on the emergence of dengue epidemics in Pakistan. PNAS 2015; 112: 11887–92.

9 Romeo-Aznar V, Picinini Freitas L, Gonçalves Cruz O, King AA, Pascual M. Fine-scale heterogeneity in population density predicts wave dynamics in dengue epidemics. Nat Commun 2022; 13: 996.

10 Franklinos LHV, Jones KE, Redding DW, Abubakar I. The effect of global change on mosquito-borne disease. The Lancet Infectious Diseases 2019; 19: e302–12.

11 Oidtman RJ, Lai S, Huang Z, et al. Inter-annual variation in seasonal dengue epidemics driven by multiple interacting factors in Guangzhou, China. Nat Commun 2019; 10: 1148.

12 Xu L, Schmid BV, Liu J, Si X, Stenseth NC, Zhang Z. The trophic responses of two different rodent-vector-plague systems to climate change. Proc R Soc B-Biol Sci 2015; 282: 20141846.

13 Feng Y, Fan M, Gao Y, et al. Epidemiological features of four human plague cases in the Inner Mongolia Autonomous Region, China in 2019. Biosafety and Health 2020; 2: 44–8.

14 Li J, Wang Y, Liu F, et al. Genetic source tracking of human plague cases in Inner Mongolia-Beijing, 2019. PLOS Neglected Tropical Diseases 2021; 15: e0009558.

15 Zhang Z, Li Z, Tao Y, et al. Relationship between increase rate of human plague in China and global climate index as revealed by cross-spectral and cross-wavelet analyses. Integr Zool 2007; 2: 144–53.

16 Yuan X, Yang L, Li H, Wang L. Spatiotemporal Variations of Plague Risk in the Tibetan Plateau from 1954–2016. Biology 2022; 11: 304.

17 Kovats RS, Bouma MJ, Hajat S, Worrall E, Haines A. El Niño and health. The Lancet 2003; 362: 1481–9.

18 Yan J, Zhang T, Gong S. Spatio-temporal distribution and environmental influencing factors of cholera in Sichuan Province during the Republic of China. Journal of Central China Normal University (Natural Sciences) 2023; : 1–12.

19 Zoua W, Djaouda M, Maïworé J, Liang S, Nola M. Scarcity of Potable Water and Sanitation Facilities in the Endemic Cholera Region of North Cameroon. Journal of Environment Pollution and Human Health 2019; 8: 6–19.

20 Zinsstag J, Schelling E, Waltner-Toews D, Tanner M. From “one medicine” to “one health” and systemic approaches to health and well-being. Preventive Veterinary Medicine 2011; 101: 148–56.

# Box S1. Case Study: 2023 Typhoon Doksuri

| The summer of 2023 in Beijing and neighboring Hebei and Tianjin areas have been battered by severe climate events, with the city grappling with a historically severe heatwave followed by a lethal flash flood due to Typhoon Doksuri. The confluence of these weather events underscores the mounting threat of climate change and the urgent need for effective public health responses.  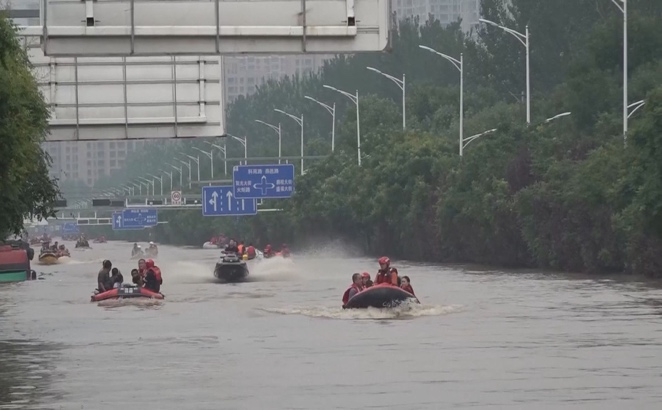Heavy rainfall, with record-breaking precipitation levels in some areas, triggered severe flooding, landslides, and disasters. Beijing and surrounding areas saw flood water leading to death tolls and missing people, washed away cars, crippled spillway bridges, drowned underground parking structures, and led to devastating economic damage to storage supplies such as valuable books, life savings, and assets. Boats now roam on what used to be roads. Emergency response and rescue efforts have been ongoing. The authorities scrambled to support affected areas. Over 100,000 people were evacuated in Beijing, with 11 reported deaths recorded there and another nine in neighboring Hebei Province ^1^. Authorities had issued a red alert, cautioning that this was the heaviest deluge witnessed in over 70 years.  Immediately before the flood, the city had already endured its highest recorded temperatures, surpassing 40 degrees Celsius (104 degrees Fahrenheit). The severity of the heat has been so extraordinary that national weather stations in Beijing and Hebei registered the hottest temperature for July ever recorded. Oppressive heat and meteorological red alerts, advise suspending outdoor work and measures to prevent heatstroke. Extreme temperatures, especially when prolonged, will have wide-reaching impacts on everyday life, from straining power grids as the demand for air-conditioning soars to the demand for sunproof products. |
| --- |

# Box S2. Case Study: Climate Change Adaptation and Health Implications in Shenzhen's Urban Development

Located opposite Hong Kong, Shenzhen's subtropical climate and urbanized landscape intensify its vulnerability to prolonged, oppressive heatwaves, further accentuating the urgency for climate-resilient infrastructure to protect public health from these and other climate change-induced threats. Shenzhen, famous for its swift urban growth and technological prowess, is acutely aware of the health and environmental challenges posed by climate change. As part of its adaptation efforts, the city has invested significantly in green building infrastructure. By 2021, over 160 million square meters were dedicated to green building construction, comprising more than 1,500 projects, with the overarching objective of mitigating the urban heat island effect and enhancing overall air quality. This commitment to sustainable construction directly influences public health by creating cooler urban environments and reducing pollution levels.

Green spaces, known to positively impact mental and physical health, have been a focal point in Shenzhen's urban planning. By the end of 2021, the city had established 1,238 diverse parks and introduced the "Park City Planning Outline". The ambitious goal set for 2035 aims for 2,000 citywide parks, 2,000 community co-built gardens, and 500 kilometers of suburban paths, aiming to provide residents with ample green spaces for recreation and to combat the effects of heat waves.

Shenzhen has introduced innovations like the District Cooling System (DCS) to address rising urban temperatures, enhancing comfort in public spaces and mitigating heat-related health risks. Alongside these measures, Shenzhen utilizes the Internet of Things (IoT) to create a more responsive urban environment. Being a hub of technological advancement in China, the city employs IoT devices for real-time monitoring of environmental factors, including air quality and temperature. This data guides effective strategies for climate challenges, such as using IoT to alleviate traffic congestion and reduce emissions. Furthermore, smart grids and building sensors optimize energy use. Through these integrated measures, Shenzhen advances its urban management while addressing climate and health-related issues.

| 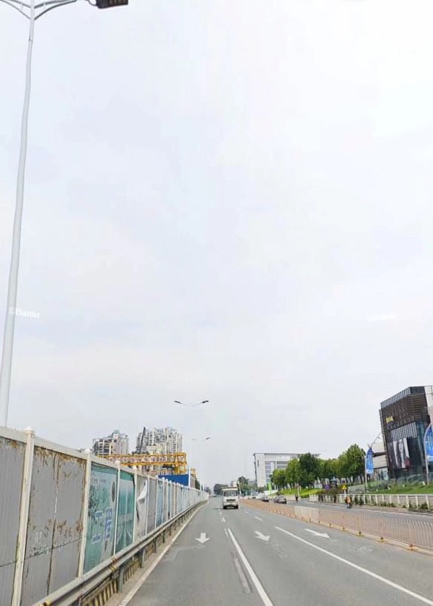 | 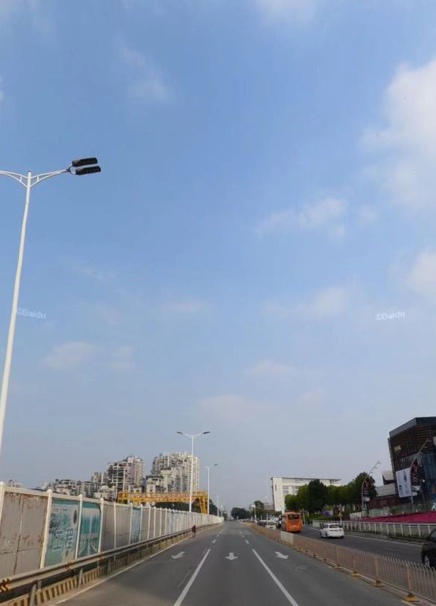 | 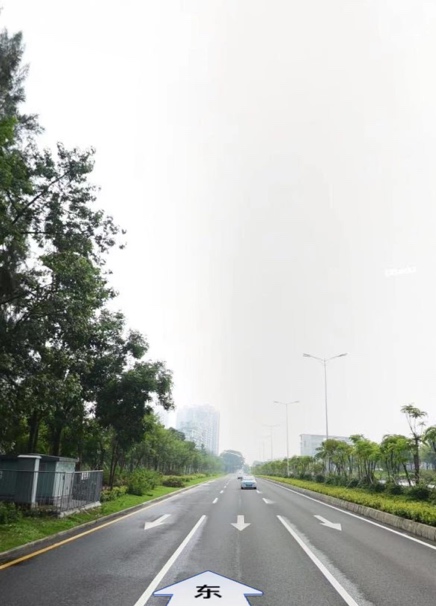 |
| --- | --- | --- |

Caption: Time Series Changes in the visibility of green spaces on Binhai Avenue, Shenzhen

**
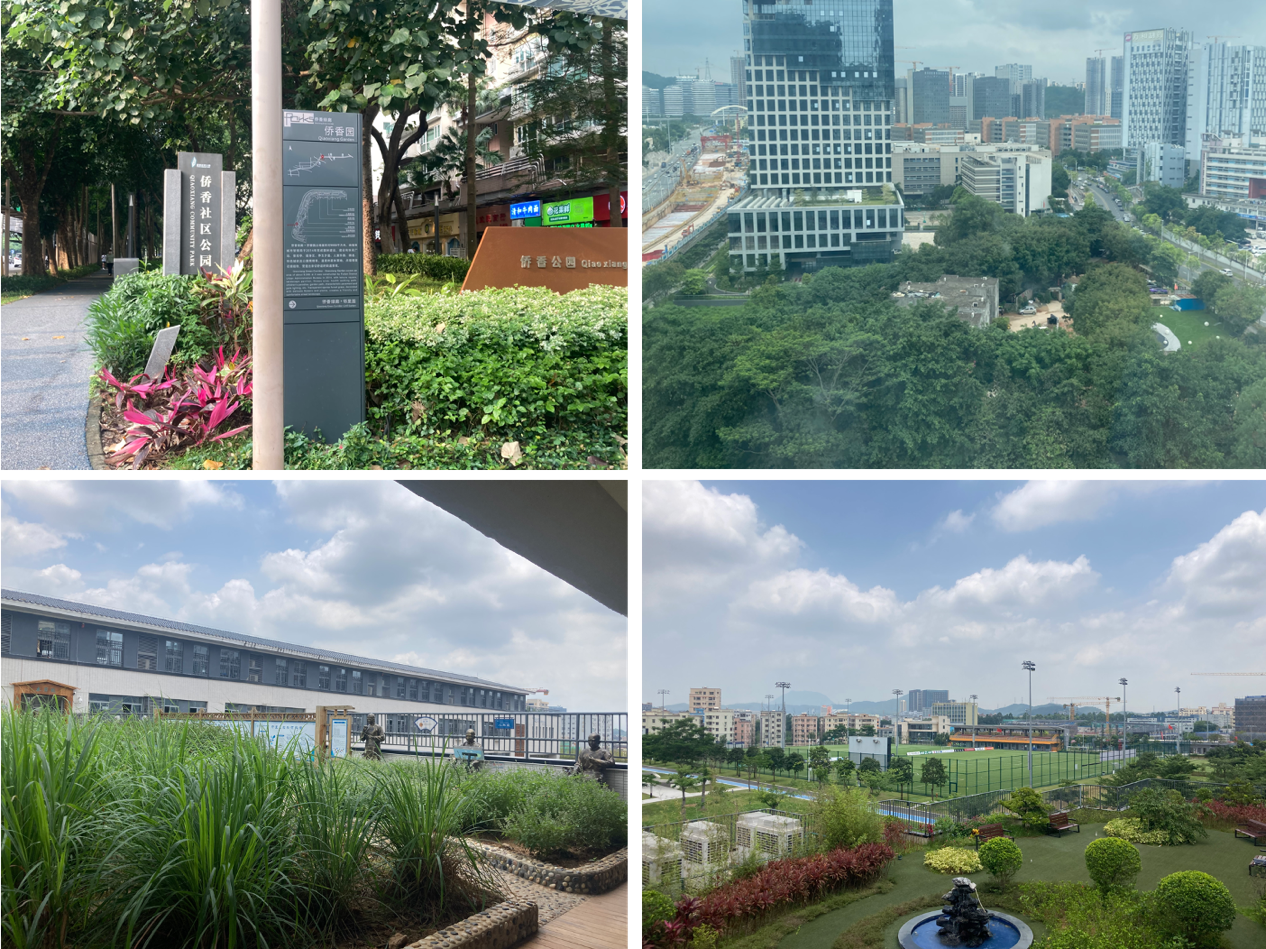
**

Caption: Parks and schools in Shenzhen

# Box S3. Case Study: Shenzhen's dedication to eco-friendly and health-centric urban development

The Futian Central Business District (CBD), often referred to as "China's Manhattan", showcases Shenzhen's dedication to eco-friendly and health-centric urban development. Beyond its architectural aesthetics, the CBD's design emphasizes green construction, including a solar photovoltaic system. This system not only reduces carbon emissions but also modulates building temperatures, which has potential implications for reduced energy consumption, especially during extreme heat events. However, a comprehensive assessment of the building's total energy footprint is pivotal to determining its actual environmental and health-related benefits.

**
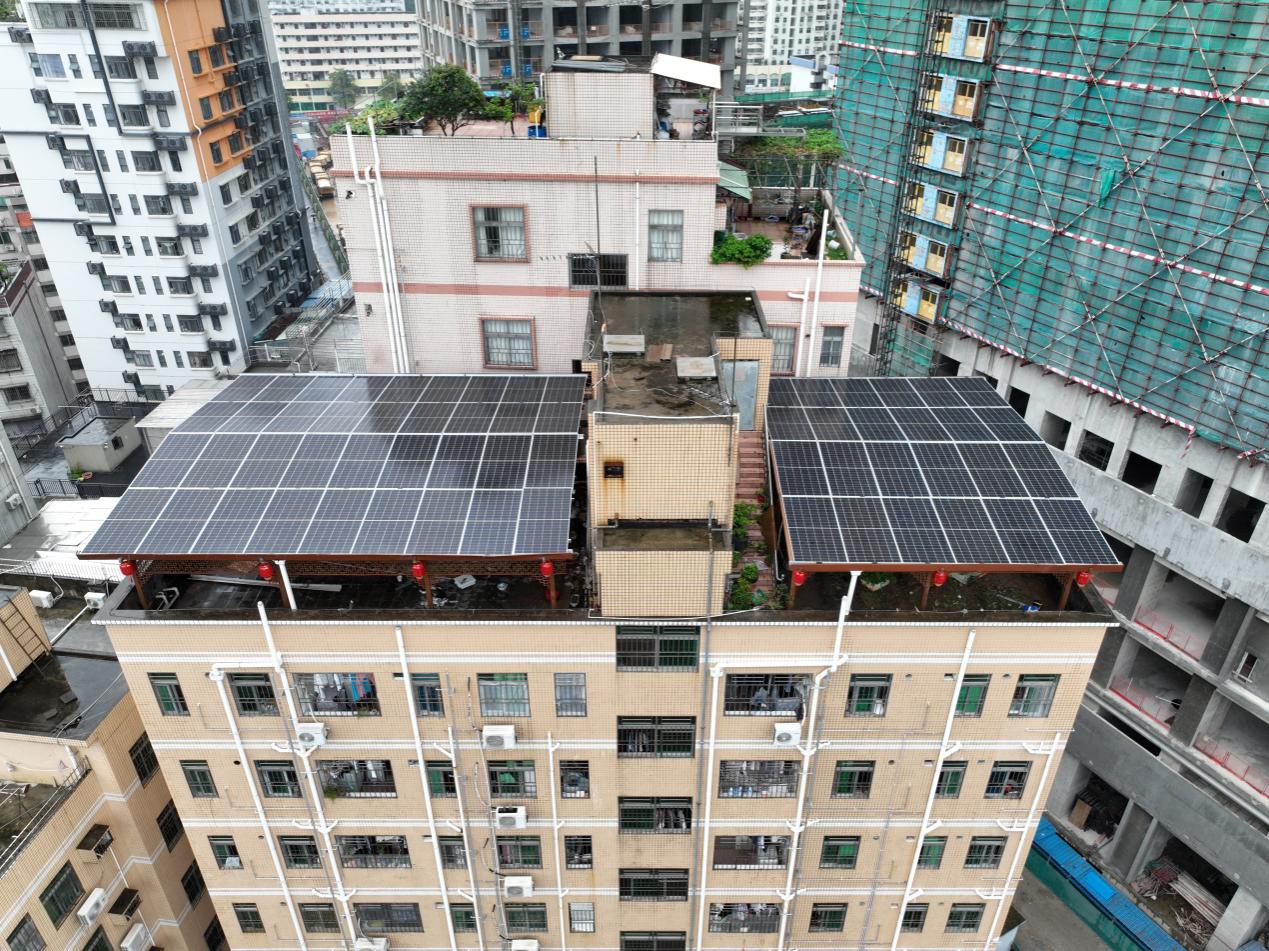
**

Caption: In Shenzhen's Futian district, a distributed photovoltaic (PV) project with a capacity of 3MW has been initiated in the Shangmeilin village. The pilot project, with an installed capacity of 32.7kW, produces around 34,000 kWh annually, resulting in an estimated carbon reduction of 11.2 tons. This urban village PV upgrade architectural design utilizes that offers rooftop heat shielding and creates functional outdoor spaces.
